# Dextromethorphan Dampens Neonatal Astrocyte Activation and Endoplasmic Reticulum Stress Induced by Prenatal Exposure to Buprenorphine

**DOI:** 10.1155/2021/6301458

**Published:** 2021-07-10

**Authors:** Chun-Hua Lin, Pao-Luh Tao, Huey-Jen Tsay, Yao-Chang Chiang, Wei-Tang Chang, Ing-Kang Ho, Feng-Shiun Shie

**Affiliations:** ^1^Department of Nursing, College of Nursing and Health, Kang-Ning University, Taipei, Taiwan; ^2^Center for Neuropsychiatric Research, National Health Research Institutes, Zhunan Town, Miaoli County, Taiwan; ^3^Institute of Neuroscience, National Yang-Ming University, Taipei, Taiwan

## Abstract

Prenatal exposure to buprenorphine renders offspring vulnerable to cerebral impairments. In this study, our data demonstrate, for the first time, that prenatal exposure to buprenorphine escalates astrocyte activation concurrent with indications of endoplasmic reticulum (ER) stress in the hippocampi of neonates, and this can be prevented by the coadministration of dextromethorphan with buprenorphine. Furthermore, dextromethorphan can inhibit the accumulation of GPR37 in the hippocampus of newborns caused by buprenorphine and is accompanied by the proapoptotic ER stress response that involves the procaspase-3/CHOP pathway. Primary astrocyte cultures derived from the neonates of the buprenorphine group also displayed aberrant ER calcium mobilization and elevated basal levels of cyclooxygenase-2 (COX-2) at 14 days *in vitro* while showing sensitivity to lipopolysaccharide-activated expression of COX-2. Similarly, these long-lasting defects in the hippocampus and astrocytes were abolished by dextromethorphan. Our findings suggest that prenatal exposure to buprenorphine might instigate long-lasting effects on hippocampal and astrocytic functions. The beneficial effects of prenatal coadministration of dextromethorphan might be, at least in part, attributed to its properties in attenuating astrocyte activation and hippocampal ER stress in neonates.

## 1. Introduction

Because a growing number of pregnant opioid users receive buprenorphine maintenance therapy, concerns regarding the adverse effects of this long-lasting opioid on the brain function of offspring have received increasing attention [1–4]. Indeed, visuomotor dysfunction has been reported in children prenatally exposed to opioids [5]. In neonatal animal models, offspring with prenatal exposure to buprenorphine have several brain dysfunctions, including demyelination, abnormal cholinergic/dopaminergic development, and decreased neurogenesis; such exposure may also increase the death rate in neonates [6–9]. The mechanisms underlying the adverse effects of prenatal exposure to buprenorphine on the brain function are not fully understood although *μ*-opioid receptor-mediated neurotoxicity in adults has been proposed [10].

Given the significant roles of astrocytic function in pathophysiological conditions, we propose that dysfunctional astrocytes and associated oxidative and endoplasmic reticulum (ER) stress might contribute to the development of brain dysfunction triggered by the prenatal exposure to opioids. Astrocytes, which are the most abundant glia in the brain, play an important role in synaptic information processing by balancing both excitatory glutamatergic and inhibitory GABAergic neurotransmission, particularly where pre- and postsynaptic nerve endings are intricately wrapped by astrocytes [11, 12]. Astrocytic function and astrocyte-neuron intercellular communication are regulated largely by the homeostasis of intracellular calcium [13]. In many brain diseases, astrocytes are known to be critical for neuronal survival. Astrocytes become activated with morphological changes and increased expression of glial fibrillary acidic protein (GFAP) during the pathogenesis of brain diseases [14]. Similar to microglial activation, astrogliosis is a common pathological feature shared by many brain diseases. Although astrocyte activation has been proposed to promote neuronal survival in the diseased brain, uncontrolled release of effectors derived from overactivated astrocytes triggers neuroinflammation and increases oxidative and ER stresses [15]. Indeed, proinflammatory cytokines secreted from astrogliosis in neurodegenerative diseases could compromise glutamatergic neurotransmission and synaptic activity through disrupting the interaction of postsynaptic density protein 95 with N-methyl-D-aspartate receptors (NMDARs) [16]. These consequences lead to enhanced excitotoxicity in the brain and, ultimately, loss of neuronal function.

Increased expressions of caspase-3 and C/EBP homologous protein (CHOP) are indicative of proapoptosis during ER stress [16, 17]. Other ER stress proteins and chaperones such as glucose-regulated protein (GRP) and protein-disulfide isomerase (PDI) are produced in response to ER stress in order to attenuate the accumulation of mutated gene products or misfolded proteins induced by oxidative or nitrosative stress [18]. Expression of PDI facilitates the maturation and transport of misfolded proteins, representing an adaptive response to prevent ER-associated neurotoxicity and protein misfolding [19]. Reduced PDI expression [20] or inhibition of PDI activity [18] results in aberrant ER function. G protein-coupled receptor 37 (GPR37) is an orphan G protein-coupled receptor that is expressed in the brain and is associated with neuropathology of Parkinson's disease (PD) [21, 22]. Recently, accumulation of GPR37 aggregates has been considered to result from protein misfolding, which is characteristic of ER stress [23]. GPR37 is a substrate for parkin involved in the modulation of dopaminergic signaling [24], while increasing expression and accumulation of GPR37 high-molecular-weight aggregates instigate ER stress [25]. Furthermore, the elevated expression of GPR37 has been shown to lead to protein aggregation, thus contributing to neuronal dysfunction [26, 27]. Oxidative stress refers to the production of large amounts of reactive oxygen species, such as NO derivatives, and proinflammatory cytokine/chemokines and prostanoids, which are associated with an increased activity of cyclooxygenase-2 (COX-2) and inducible nitric oxide synthase (iNOS) [28]. These effectors may directly damage neuronal function and/or lead to uncontrolled glial overactivation, amplifying a self-propelling cycle that potentiates further increase in oxidative and ER stresses [29]. The consequences include massive neuroinflammation and exacerbated neuronal dysfunction.

Dextromethorphan, a common ingredient in cough and cold remedies, has been documented to show anti-inflammatory activity and NMDAR-dependent antagonistic effects on neuroprotection in many disease models, such as lipopolysaccharide- (LPS-) induced neurodegeneration, Parkinson's disease [27, 30], seizures [31, 32], and ischemic brain injury [33]. The mechanism underlying the neuroprotective effects of dextromethorphan has been proposed to be through the reduction of NMDAR-mediated excitotoxicity, although alternative targets have also been reported [34, 35]. Dextromethorphan also modulates the behavioral responses induced by several substance abuse drugs. For instance, dextromethorphan attenuates methamphetamine-induced rewarding as determined by conditioned place preference [36]. Therefore, as a result of its influence on the expression of neurotrophic effectors and its role in modulating glial-activation-associated neuroinflammation, we speculate that coadministration of dextromethorphan can be beneficial for offspring with prenatal exposure to buprenorphine. Our study on the therapeutic potential of dextromethorphan may provide findings that could significantly contribute to the knowledge of this important public issue in drug abuse medicine.

## 2. Materials and Methods

### 2.1. Animals

Pregnant Sprague-Dawley (SD) rats were maintained at a room temperature of 23°C ± 2°C, with a 12 h light-dark cycle and food and water *ad libitum*, throughout the experiment. Animals were handled according to the guidelines of the Institutional Animal Care and Use Committee at the National Health Research Institutes. The rats were randomly separated into the following four groups: subcutaneous injection of water (vehicle control group), buprenorphine, dextromethorphan, or buprenorphine plus dextromethorphan from embryonic day E3 to E20. Buprenorphine was delivered at 3 mg/kg once a day, while dextromethorphan was delivered at the same dose (3 mg/kg) twice a day (9 a.m. and 5 p.m.). Neonates at postnatal day 1 (P1) were used for all assessments, while offspring at postnatal day 14 were used for the hippocampal synaptic assay. Neonates in the same litter were considered identical, and the value of *n* represents the number of pregnant SD rats used in the experiments.

### 2.2. Primary Astrocyte Cultures

Primary astrocyte cultured cells were derived from cortices of P1 neonates with different prenatal treatments; the primary astrocyte cultures were performed as described previously [37]. Briefly, cells were dissociated using an enzyme solution containing Dulbecco's modified Eagle's medium (DMEM), ethylenediaminetetraacetic acid (0.5 mmol/L), L-cysteine (0.2 mg/mL), papain (15 U/mL), and DNase I (200 *μ*g/mL), followed by trituration. Culture medium (DMEM with 10% fetal bovine serum, 100 U/mL penicillin, and 100 *μ*g/mL streptomycin) was changed after 2 h of initial seeding. After two stripping and seeding cycles during two weeks in culture, astrocytes at 14 days *in vitro* (DIV) were harvested with trypsinization and reseeded for the experiments.

### 2.3. Confocal Microscopy

Confocal microscopy was used for the semiquantification analysis. Brain samples from P1 neonates were subjected to paraformaldehyde (4% in PBS) fixation overnight, followed by cryoprotection with sucrose (30% in PBS). One representative 30 *μ*m thick cryosection per neonate from the control or buprenorphine group (at least four litters) was subjected to immunohistochemical analysis using antibodies against GFAP (Millipore), GPR37 (Abnova), or PDI (Santa Cruz). Images were acquired with a 20x objective using a Leica confocal microscopy imaging system with identical settings for the parameters, while a 63x objective was used for images of CA1 at a higher magnification. Mounting medium (Vector Lab) containing 4,6-diamidino-2-phenylindole (DAPI) was used to label the nuclei. Semiquantification of the immunoreactivity was performed using ImageJ in the fields of CA1 or hippocampal fissure, and comparisons of the mean intensity of immunoreactivity between control and buprenorphine groups in a given area were performed via *t*-test. Representative images from each group were taken for assessment.

### 2.4. Western Blot Analysis

Lysates from the hippocampus or primary culture were prepared using a lysis buffer (50 mM Tris, 150 mM NaCl, 0.02% NaN3, 0.5% Nonidet P-40, 0.5% Triton X-100, and protease inhibitor mixture), and protein concentrations were determined using the Bio-Rad Protein Assay (Bio-Rad, Hercules, CA). Samples were subjected to 7.5% Tris-HCl, sodium dodecyl sulfate-polyacrylamide gel electrophoresis (SDS-PAGE), and western blot analysis, using antibodies against iNOS (BD Biosciences), COX-2 (BD Biosciences), GFAP (Millipore), GPR37 (Abnova), GRP78 (Novus), procaspase-3 (Stressgen), caspase-3 (Cell Signaling), caspase-12 (Stressgen), and CHOP (Abcam). An antibody against *β*-actin (Novus) was used to normalize the quantity of protein in each sample. Enhanced chemiluminescence (ECL; PerkinElmer) was used for the western blot detection. Corresponding bands were quantified with ImageJ (NIH imaging software), and one-way ANOVA was tested for significance. Data are presented as the mean ± SEM (the standard error of the mean). At least four litters from four pregnant rats of each group were used for all assessments.

### 2.5. FLIPR Calcium Assay

To measure calcium transients, primary astrocytes at 14 DIV were seeded initially at 4 × 10^4^ cells per well in 96-well plates (black with a clear flat bottom from Corning) and cultured for 48 h. The assay was performed using a FLIPR Calcium 4 assay kit (Molecular Devices) according to the manufacturer's instructions. Briefly, 1 h prior to performing the assay, the culture medium was replaced with serum-free DMEM, followed by 1 h incubation at 37°C with FLIPR calcium assay reagent dissolved in 1x assay buffer containing HBSS (5 mM KCl, 0.3 mM KH_2_PO_4_, 138 mM NaCl, 4 mM NaHCO_3_, 0.3 mM Na_2_HPO_4_, 5.6 mM d-glucose, and 20 mM HEPES, pH 7.4 from Gibco) and 2.5 mM probenecid in the presence or the absence of 1.27 mM CaCl_2_. After stabilization at room temperature for 1 h, the culture plates were subjected to analysis using FlexStation 3 (Molecular Devices) with robotic addition of glutamate (final concentration of 100 *μ*M in HBSS) into the cultures at 10 sec after the initial recording. The fluorescence was monitored at every 1.52 sec interval with an excitation wavelength of 485 nm and an emission wavelength of 525 nm for 180 sec. The intracellular calcium reached a peak at approximately 13 sec after the induction of glutamate. Data were analyzed using SoftMax Pro (Molecular Devices) and are presented as a response curve and an area under the curve normalized by the corresponding area at the basal condition. Data from duplicated experiments in two wells of each culture were averaged, and at least five litters from five pregnant rats of each group were used for quantification.

### 2.6. Statistical Analysis

The differences between groups were tested using one-way ANOVA followed by post hoc multiple comparisons using the Bonferroni test; assessments were done with SPSS software, while the comparisons of the immunoreactivity between control and buprenorphine groups were performed via *t*-test. Data are presented as means ± SEM. Statistical significance was set at *p* < 0.05.

## 3. Results

### 3.1. Elevated Astrocyte Activation in the Hippocampus of Neonates with Prenatal Exposure to Buprenorphine

**Figure 1:**
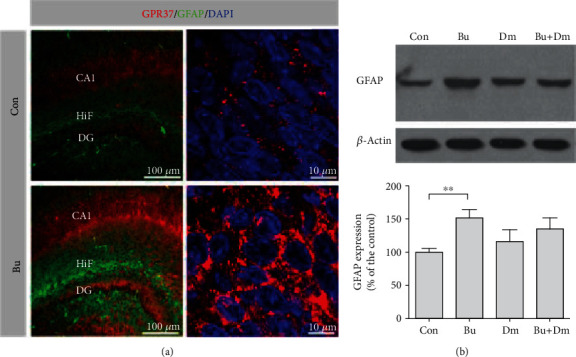
Prenatal exposure to buprenorphine escalates astrocyte activation in the hippocampus of the neonates concurrent with increased GPR37 immunoreactivity. Prenatal treatments: buprenorphine (Bu), dextromethorphan (Dm), coadministration of buprenorphine with dextromethorphan (Bu+Dm), or vehicle controls (Con). (a) Confocal images (20x objective, left panel) for GPR37 (red) and GFAP (green) immunoreactivity in the hippocampus of neonates, with images at a higher magnification (63x objective) for GPR37 punctate staining (red) in the CA1 (right panel). (b) Western blot analysis of hippocampal GFAP expression levels with quantification. DAPI (blue) staining identifies nuclei. Data are mean ± SEM % of control, ^∗∗^*p* < 0.01 vs. Con and ^∗∗∗^*p* < 0.001 vs. Con.

Previous studies have demonstrated that the GFPA protein plays a critical role and has marked upregulation in CNS injury [38, 39]. To examine whether astrocytes are activated in neonates following prenatal exposure to buprenorphine, immunofluorescent confocal microscopy was performed using an antibody against GFAP for the control and buprenorphine groups. Semiquantification of immunoreactivity demonstrated that the buprenorphine group had increased GFAP immunoreactivity, localized mainly in the hippocampal fissure (222 ± 32% of the control group, *p* < 0.01 by *t*-test), compared to the controls (100 ± 11%) (Figure 1(a)). To quantify GFAP levels as an indication of astrocyte activation, total hippocampal lysates from all groups were analyzed by western blot. Results showed that GFAP levels in the buprenorphine group were significantly increased to 152 ± 12% of the control group (*p* < 0.001; Figure 1(b)), which is suggestive of astrocyte activation. Since dextromethorphan is known to alleviate glial activation, the effects of coadministration of dextromethorphan with buprenorphine were examined. Intriguingly, the levels of the hippocampal GFAP in the group with prenatal coadministration of dextromethorphan with buprenorphine returned to those (127 ± 16% of the control group) similar to what is seen in the controls (100 ± 6%), while the dextromethorphan-alone group (116 ± 18% of the control group) showed no significant effect on GFAP levels.

### 3.2. Prenatal Exposure to Buprenorphine Leads to Increased ER and Oxidative Stress in the Hippocampus of Neonates

**Figure 2:**
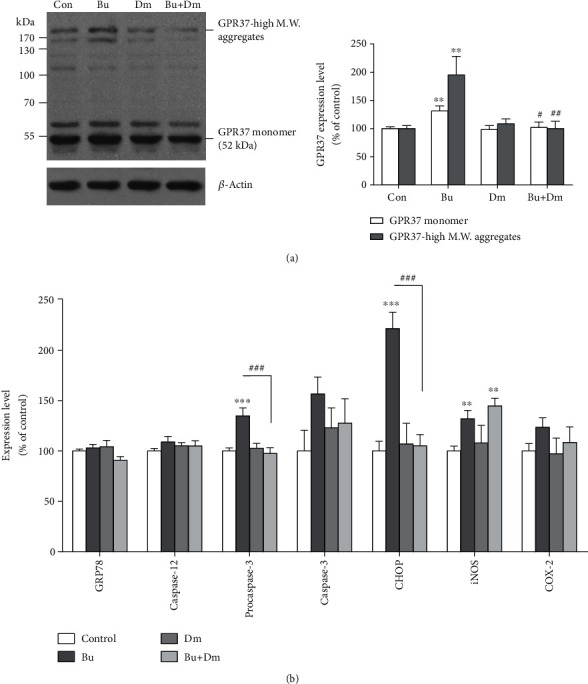
ER and oxidative stresses are induced by prenatal exposure to buprenorphine in the hippocampus of the neonates. (a) Western blot analysis of the expression levels of hippocampal GPR37 monomer and high molecular weight (M.W.) aggregates with quantification. (b) Quantification of Western blot analysis for the expression levels of GPR78, caspase-12, procaspase-3, caspase-3, CHOP, iNOS, and COX-2 in the hippocampus of the neonates with prenatal exposure to Bu, Dm, Bu+Dm, or Con. Data are mean ± SEM % of control; ^∗∗^*p* < 0.01 vs. Con and ^∗∗∗^*p* < 0.001 vs. Con and ^##^*p* < 0.01 vs. Bu and ^###^*p* < 0.001 vs. Bu.

Given that the accumulation of GPR37 aggregates is indicative of ER stress [22], brain tissues were then subjected to analysis under immunofluorescent confocal microscopy. The data show that the increasing GFAP immunoreactivity in the hippocampal fissure of the buprenorphine group parallels the intensity of the GPR37 punctate in the pyramidal neurons (Figure 1(a)). Results from immunohistochemistry clearly showed that GPR37 immunoreactivity was predominantly present in the pyramidal neurons of areas CA1-3 and, to a lesser extent, in the granules of the dentate gyrus (DG). As demonstrated in representative images at a higher magnification in CA1, semiquantification of immunoreactivity shows that perinuclear GPR37 punctate staining was significantly increased in the group with prenatal exposure to buprenorphine compared to the controls. Quantification of GPR37 aggregates by western blot revealed that buprenorphine exposure led to an increase in GPR37 accumulation in both monomer (at 52 kDa; 131 ± 9% of the control group, *p* < 0.01) and high-molecular-weight aggregates (>170 kDa; 195 ± 33% of the control group, *p* < 0.01) compared to controls (100 ± 3% and 100 ± 5%, respectively; Figure 2(a)). The group with coadministration of dextromethorphan with buprenorphine showed reversal in the induction of GPR37 expression (102 ± 9% of the control group) and prevention of the accumulation of GPR37 high-molecular-weight aggregates (100% ± 13% of the control group), while the dextromethorphan-alone group (98 ± 7% and 109 ± 9% of the control group for monomer and high-molecular-weight aggregates, respectively) showed no changes in GPR37 levels.

Quantification of other indications of the prosurvival and proapoptotic pathways of ER stress responses from western blot analysis is presented in Figure 2(b). Data showed that the expression levels of procaspase-3 and CHOP (135 ± 8% and 221 ± 16% of the control group, respectively) were significantly increased (*p* < 0.001 and *p* < 0.01, respectively) in the hippocampus of neonates with prenatal exposure to buprenorphine compared to that of the controls (100 ± 3% and 100 ± 10%, respectively). Intriguingly, coadministration of dextromethorphan with buprenorphine reversed the induction of procaspase-3 and CHOP expressions (100 ± 6% and 105 ± 11% of the control group, respectively). Levels of caspase-3 appear to be higher in the buprenorphine group (157 ± 17%) than in other groups, although not significantly. However, the expression levels of GRP78 and caspase-12 were comparable among all groups of prenatal treatments. Taken together, these data demonstrate that prenatal exposure to buprenorphine led to the accumulation of hippocampal GPR37 aggregates in the hippocampus of the neonates concurrent with proapoptotic ER stress responses involved in the procaspase-3/CHOP pathways, which is rescued by the coadministration of dextromethorphan.

For measuring oxidative stress, expression levels of iNOS and COX-2 in the hippocampus were examined. Results from the western blot analysis showed that the expression levels of iNOS were significantly increased in the buprenorphine group (132 ± 8% of the control group, *p* < 0.01) compared to the control group (100 ± 5%). The expression levels of iNOS were not affected by dextromethorphan alone (97 ± 16% of the control group), while the group with coadministration of dextromethorphan and buprenorphine showed no reversal in the induced iNOS expression (146 ± 8% of the control group, *p* < 0.01). The expression levels of COX-2 were slightly increased in the buprenorphine group (124 ± 9% of the control group), although not significantly, while those in the dextromethorphan and buprenorphine and coadministration of dextromethorphan groups were 97 ± 16% and 108 ± 15% of the control level, respectively. These were both not significantly different from the levels in the control group (100 ± 7%). Our data demonstrate that the prenatal exposure to buprenorphine induces oxidative stress as evidenced by the elevated iNOS levels in the hippocampus. However, coadministration of dextromethorphan with buprenorphine had minimal effects on preventing induced oxidative stress.

### 3.3. Prenatal Exposure to Buprenorphine Increases the Levels of COX-2 Expression at Both Basal and LPS-Activated States in Primary Astrocytes

**Figure 3:**
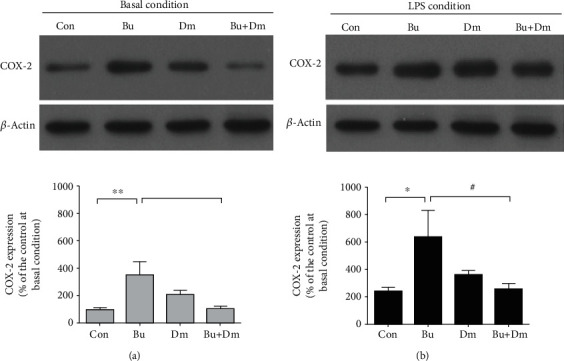
Primary astrocytes derived from the neonates of the buprenorphine group display elevated basal levels of COX-2 and sensitize to the LPS-induced expression of COX-2. Western blot analysis with quantification for COX-2 expression levels in the primary astrocytes at basal (a) or activated (LPS treatment) (b) condition. Data are mean ± SEM % of control; ^∗^*p* < 0.05 vs. Con and ^∗∗^*p* < 0.01 vs. Con and ^##^*p* < 0.001 vs. Bu.

Given that astrocytes play an important role in hippocampal functioning [40], the observed long-term effects *in vivo* can be indicative of abnormal astrocytic functions induced by prenatal exposure to buprenorphine. Thus, we first investigated whether prenatal exposure to buprenorphine causes long-term changes in astrocytic immunity. Expressions of COX-2 and iNOS were measured in the primary astrocytes derived from neonates subjected to the various prenatal treatments. As presented in Figure 3(a), the basal levels of the COX-2 expression were higher in astrocytes in the group with prenatal exposure to buprenorphine (388 ± 109% of the control group at basal condition, *p* < 0.01) compared to the control group (100 ± 10%). Data suggest that buprenorphine-induced oxidative stress in astrocytes is sustained at 14 DIV. Surprisingly, astrocytes from the group with prenatal exposure to dextromethorphan alone also had increased basal levels of COX-2 expression (211 ± 28% of the control group at the basal condition), although not significantly. Coadministration of dextromethorphan with buprenorphine reduced the expression of COX-2 to levels (102 ± 20% of the control group at the basal condition) comparable to those in the controls.

We then investigated whether prenatal exposure to buprenorphine sensitizes astrocytes to a greater induction of COX-2 expression under the circumstances of an immune challenge. As presented in Figure 3(b) for the control group, expression levels of COX-2 in primary astrocytes can be significantly elevated by LPS treatments (241 ± 29% of the control group at the basal condition). The levels of COX-2 were further enhanced in primary astrocytes derived from the buprenorphine group (640 ± 195% of the control group at basal condition). Levels of LPS-induced COX-2 expression in groups with the administration of dextromethorphan alone and coadministration of dextromethorphan with buprenorphine were 364 ± 26% and 259 ± 41% of the control group at the basal condition, respectively, which were significantly higher than those of the control group at basal conditions but not higher than those of the control group that received LPS treatments. Thus, coadministration of dextromethorphan with buprenorphine appears to reverse the effects of buprenorphine on the regulation of astrocytic immunity.

Next, we examined the iNOS expression, which is another indication of immune response, in astrocytes. Since the basal levels of iNOS in the primary astrocytes were undetectable by western blot, data from LPS-activated astrocytes are presented. Our results show that the LPS-induced expressions of iNOS are comparable among all groups; the levels in the control, buprenorphine, dextromethorphan, and coadministration of dextromethorphan with buprenorphine groups were 100 ± 18%, 82 ± 6%, 99 ± 18%, and 112 ± 24% of the control group with LPS treatment, respectively. Data suggest that coadministration of dextromethorphan with buprenorphine had minimal effects on preventing the induced iNOS expression.

### 3.4. ER Calcium Mobilization in Response to Glutamate in Primary Astrocytes at 14 DIV Is Altered by Prenatal Exposure to Buprenorphine

**Figure 4:**
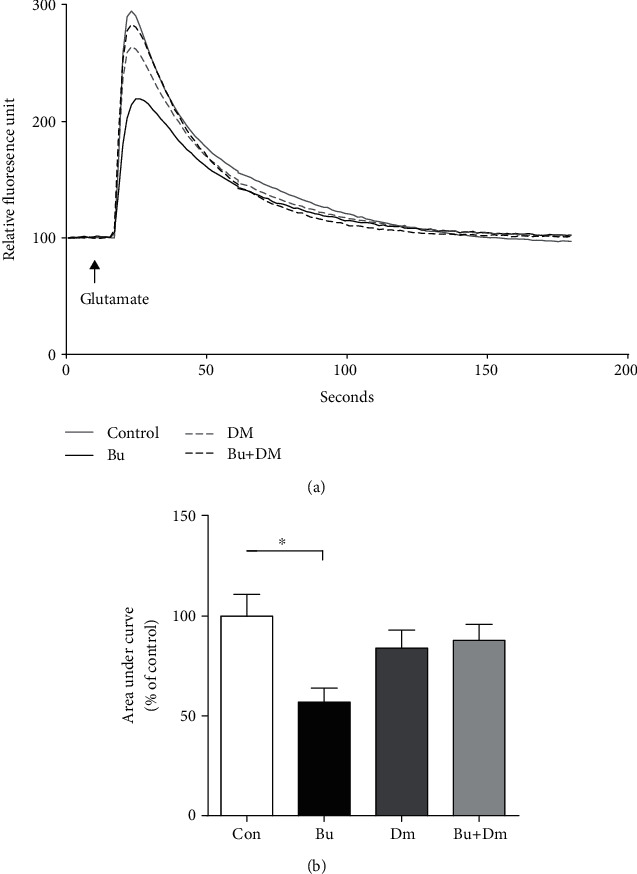
Glutamate-induced calcium transient is inhibited in the primary astrocytes from the neonates with prenatal exposure to buprenorphine. Response curve and quantification of calcium transients in the absence of extracellular calcium measured by the FLIPR calcium assay using FlexStation 3 in the primary astrocytes from neonates with prenatal exposure to Bu, Dm, Bu+Dm, or Con. Addition of glutamate (arrow) at 10 sec after the initial recording. Data are mean ± SEM % of control; ^∗^*p* < 0.05 vs. Con.

Next, we investigated whether glutamate-induced ER calcium mobilization, a critical process for modulating astrocytic function in response to immune challenges in primary astrocytes, is affected by prenatal exposure to buprenorphine at 14 DIV. As shown in Figure 4, glutamate-induced transient reduction in calcium in the absence of extracellular calcium is observed in the astrocytes of the group with prenatal exposure to buprenorphine (57 ± 7% of the control group, *p* < 0.05) compared to the control group (100 ± 11%), which is suggestive of a defect in calcium mobilization from ER stores. Coadministration of dextromethorphan with buprenorphine reversed the glutamate-induced transient reduction in calcium to an extent (84 ± 9% of the control group) that was not significantly different from those in controls, while the dextromethorphan group alone had no effect on the glutamate-induced transient reduction in calcium in astrocytes (88 ± 8% of the control group). Our data show that prenatal exposure to buprenorphine caused deficits in calcium mobilization from the ER in primary astrocytes, while coadministration of dextromethorphan completely abolished the effects. The magnitude of glutamate-induced transient reduction in total intracellular calcium as measured in the presence of extracellular calcium for all groups, including buprenorphine (105 ± 8%), dextromethorphan (82 ± 10%), and dextromethorphan-buprenorphine coadministration (96 ± 17%), was comparable to that in the controls (100 ± 7%). Thus, these data suggest that prenatal exposure to buprenorphine leads to long-term but reversible changes in astrocytic calcium mobilization by dampening ER store release while enhancing calcium influx from an extracellular source.

## 4. Discussion

Although buprenorphine replacement therapy has been considered safe for pregnant women, alterations in the brain physiology of offspring in animal models have been documented. Our results reveal the first demonstration that prenatal exposure to buprenorphine could lead to an aberrant astrocyte activation and elevated oxidative and ER stresses in the hippocampus of neonates. Furthermore, our findings regarding abnormal astrocyte activation, ER dysfunction in primary cultures, and disrupted synaptic function in the hippocampus of offspring at 2 weeks of age suggest that the impacts of prenatal exposure to buprenorphine are long lasting, both *in vivo* and *in vitro*. There is mounting evidence suggesting that astrocyte activation and the associated oxidative and ER stresses contribute to the pathogenesis of several brain diseases. Our findings provide further insights into a possible mechanistic link between astrocyte-associated oxidative and ER stresses and the development of brain dysfunction in offspring following prenatal exposure to buprenorphine. However, the possibility that other factors contribute to the underlying mechanisms, especially microglia-mediated neuroinflammation, cannot be excluded.

In our animal model, the insults to the offspring may be due to the impact of direct exposure to buprenorphine during the embryonic stages, release of proinflammatory effectors from the maternal immune system in response to the repeated exposure of buprenorphine, and stresses from self-immunity and postnatal withdrawal syndrome, all of which can either lead to further damage through uncontrolled neuroinflammation or be counteracted through neuroprotective mechanisms. Indeed, astrocyte activation is a double-edged sword that can be detrimental or beneficial to neuronal survival, which is similar to the multiple functions of microglial activation in neurodegenerative diseases. In many brain diseases, activated astrocytes provide support for damaged neuronal tissue through the release of neurotrophic factors and clearance of neurotoxic substances [37]. Thus, it is conceivable to consider astrocyte activation induced by prenatal exposure to buprenorphine as a defensive response. Alternatively, prenatal exposure to buprenorphine may render the offspring vulnerable to undergoing pathological processes with subsequent insults later in life as a result of perturbed immune machinery that was epigenetically modified during the most sensitive period of the immune system establishment. Similarly, a hypothesis has been developed that prenatal exposure to particular substances predisposes neonates to certain diseases, such as Parkinson's disease and autoimmune disease [41, 42]. This notion is also echoed by our findings that prenatal exposure to buprenorphine not only elevates basal levels of oxidative stress but also potentiates LPS-stimulated oxidative stress in primary astrocytes.

Unlike that in the animal models with neurodegenerative diseases, the extent of astrocyte activation induced by prenatal exposure to buprenorphine is mostly limited to the vicinity of the hippocampal fissure. This region-specific astrocyte activation may indicate a different vulnerability of the hippocampus. Furthermore, astrocytes in the hippocampal fissure play an important role in the regulation of local blood flow in the layers of the CA1 and dentate gyrus. Increased intracerebral blood flow in response to neuronal activity is essential to ensure blood supply to active regions, and astrocytes are critical for regulating the neurovascular response via modulation of calcium signaling [43, 44], in addition to their role in boundary demarcation and serving as scaffolds for migrating neurons. Our observations of a significant increase in astrocyte activation in the hippocampal fissure and the disruption of astrocytic calcium mobilization in primary astrocytes collectively imply that the associated oxidative and ER stress may be locally instigated. The resulting consequences may lead to abnormal astrocyte-regulated neuronal activity in the susceptible regions, thus promoting neuroinflammation. This result may partially explain why increased GPR37 aggregates, which are an indication of ER stress, were largely located in the CA1 region.

Although the molecular mechanism responsible for the effects of dextromethorphan on counteracting the buprenorphine-induced ER stress remains unclear, one possible explanation is that the beneficial effects of dextromethorphan may be attributed to its anti-inflammatory and/or antiexcitotoxic properties. This speculation is supported by the reports that inhibition of NMDAR-mediated excitotoxicity in various models of neurodegeneration contributes to the function of dextromethorphan in protecting neurons from damage and in suppressing glial activation [45, 46]. Of note, dextromethorphan is a noncompetitive, but weak, NMDAR antagonist [47], while dextromethorphan and its metabolite dextrorphan are high-affinity agonists for the sigma-1 receptor, which is a novel nonopioid ER chaperon. Thus, the effects of dextromethorphan could possibly result from the activation of the sigma-1 receptor. Increasing evidence indicates that sigma-1 receptor agonists are anti-inflammatory and neuroprotective mainly via their chaperoning functions that counteract ER stress [48]. This notion echoes the beneficial effects of dextromethorphan in counteracting the proapoptotic ER stress observed in our study. In addition, stimulation by sigma-1 receptor ligands has been shown to modulate the glutamate-evoked calcium influx in synapses [48] and to ameliorate glutamate-/NMDA-evoked NO production [49, 50]. These findings raise the possibility that indirect suppression of NMDAR activity may contribute to the anti-inflammatory properties of dextromethorphan. However, our data show that the elevated expression of iNOS was not suppressed by the dextromethorphan treatment, suggesting that the iNOS expression induced by prenatal exposure to buprenorphine might be involved in alternative mechanisms independent of the NMDAR activity. Thus, our data imply that the beneficial effects of dextromethorphan in our animal model may not be exclusively due to its blockade of the NMDAR activity.

## 5. Conclusions

In conclusion, prenatal exposure of buprenorphine aroused local astrocyte activation concurrent with increases of hippocampal oxidative and ER stresses. The consequential long-term effects on astrocytic and hippocampal functions may render the offspring sensitized upon secondary challenge in their later lives. Pharmacologically inhibiting astrocyte activation and ER stress in the hippocampus by the coadministration of dextromethorphan with buprenorphine during the embryonic stages ameliorates any detrimental effects. However, it remains unknown if the effectors instigating the signaling cascades of astrocyte activation do this as a result of the insults arising from the prenatal exposure to buprenorphine and its associated oxidative and ER stresses. This merits further investigation. Further studies on the role of astrocyte activation and its reciprocal interactions with microglia and neurons in the prenatal exposure to buprenorphine may shed light on this largely unknown area in drug abuse medicine.

## Data Availability

The original data used to support the findings of this study are included within the article.

## References

[1] Lefevre E. M., Pisansky M. T., Toddes C. (2020). Interruption of continuous opioid exposure exacerbates drug-evoked adaptations in the mesolimbic dopamine system. *Neuropsychopharmacology*.

[2] Sanchez E. S., Bigbee J. W., Fobbs W., Robinson S. E., Sato-Bigbee C. (2008). Opioid addiction and pregnancy: perinatal exposure to buprenorphine affects myelination in the developing brain. *Glia*.

[3] Jones H. E., Kaltenbach K., Heil S. H. (2010). Neonatal abstinence syndrome after methadone or buprenorphine exposure. *The New England Journal of Medicine*.

[4] Moore J. N., Gastonguay M. R., Ng C. M. (2018). The pharmacokinetics and pharmacodynamics of buprenorphine in neonatal abstinence syndrome. *Clinical Pharmacology and Therapeutics*.

[5] Melinder A., Konijnenberg C., Sarfi M. (2013). Deviant smooth pursuit in preschool children exposed prenatally to methadone or buprenorphine and tobacco affects integrative visuomotor capabilities. *Addiction*.

[6] Vestal-Laborde A. A., Eschenroeder A. C., Bigbee J. W., Robinson S. E., Sato-Bigbee C. (2014). The opioid system and brain development: effects of methadone on the oligodendrocyte lineage and the early stages of myelination. *Developmental Neuroscience*.

[7] Kuribara H., Tadokoro S. (1991). The ambulation-increasing effect of buprenorphine in mice: comparison with the effect of morphine. *Arukoru kenkyu to yakubutsu izon= Japanese journal of alcohol studies & drug dependence*.

[8] Wu C. C., Hung C. J., Shen C. H. (2014). Prenatal buprenorphine exposure decreases neurogenesis in rats. *Toxicology Letters*.

[9] Chiang Y. C., Hung T. W., Ho I. K. (2014). Development of sensitization to methamphetamine in offspring prenatally exposed to morphine, methadone and buprenorphine. *Addiction Biology*.

[10] Fitting S., McRae M., Hauser K. F. (2020). Opioid and neuroHIV comorbidity – current and future perspectives. *Journal of Neuroimmune Pharmacology*.

[11] Halassa M. M., Haydon P. G. (2010). Integrated brain circuits: astrocytic networks modulate neuronal activity and behavior. *Annual Review of Physiology*.

[12] Garnier A., Vidal A., Benali H. (2016). A theoretical study on the role of astrocytic activity in neuronal hyperexcitability by a novel neuron-glia mass model. *The Journal of Mathematical Neuroscience*.

[13] Henneberger C. (2017). Does rapid and physiological astrocyte-neuron signalling amplify epileptic activity?. *The Journal of Physiology*.

[14] Zhou B., Zuo Y. X., Jiang R. T. (2019). Astrocyte morphology: diversity, plasticity, and role in neurological diseases. *CNS Neuroscience & Therapeutics*.

[15] Beggiato S., Borelli A. C., Ferraro L., Tanganelli S., Antonelli T., Tomasini M. C. (2018). Palmitoylethanolamide blunts amyloid-*β*42-induced astrocyte activation and improves neuronal survival in primary mouse cortical astrocyte-neuron co-cultures. *Journal of Alzheimer's Disease*.

[16] Jung E. S., An K., Seok Hong H., Kim J. H., Mook-Jung I. (2012). Astrocyte-originated ATP protects A*β* (1-42)-induced impairment of synaptic plasticity. *The Journal of Neuroscience*.

[17] Li Y., Guo Y., Tang J., Jiang J., Chen Z. (2014). New insights into the roles of CHOP-induced apoptosis in ER stress. *Acta biochimica et biophysica Sinica*.

[18] Uehara T., Nakamura T., Yao D. (2006). S-Nitrosylated protein-disulphide isomerase links protein misfolding to neurodegeneration. *Nature*.

[19] Lehtonen Š., Jaronen M., Vehviläinen P. (2016). Inhibition of excessive oxidative protein folding is protective in MPP(+) toxicity-induced Parkinson's disease models. *Antioxidants & Redox Signaling*.

[20] Wang S., Chen Z., Lam V. (2012). IRE1*α*-XBP1s induces PDI expression to increase MTP activity for hepatic VLDL assembly and lipid homeostasis. *Cell Metabolism*.

[21] Morató X., Luján R., López-Cano M. (2017). The Parkinson's disease-associated GPR37 receptor interacts with striatal adenosine A_2A_ receptor controlling its cell surface expression and function in vivo. *Scientific Reports*.

[22] Marazziti D., di Pietro C., Golini E., Mandillo S., Matteoni R., Tocchini-Valentini G. P. (2009). Induction of macroautophagy by overexpression of the Parkinson's disease-associated GPR37 receptor. *The FASEB Journal*.

[23] Imai Y., Soda M., Hatakeyama S. (2002). CHIP is associated with Parkin, a gene responsible for familial Parkinson's disease, and enhances its ubiquitin ligase activity. *Molecular Cell*.

[24] Rial D., Morató X., Real J. I. (2017). Parkinson's disease-associated GPR37 receptor regulates cocaine-mediated synaptic depression in corticostriatal synapses. *Neuroscience Letters*.

[25] Leinartaité L., Svenningsson P. (2017). Folding underlies bidirectional role of GPR37/Pael-R in Parkinson disease. *Trends in Pharmacological Sciences*.

[26] Kaneko M. (2016). Physiological roles of ubiquitin ligases related to the endoplasmic reticulum. *Yakugaku Zasshi*.

[27] Hattori N., Mizuno Y. (2017). Twenty years since the discovery of the parkin gene. *Journal of Neural Transmission*.

[28] Chung H. Y., Cesari M., Anton S. (2009). Molecular inflammation: underpinnings of aging and age-related diseases. *Ageing Research Reviews*.

[29] Rojo A. I., McBean G., Cindric M. (2014). Redox control of microglial function: molecular mechanisms and functional significance. *Antioxidants & Redox Signaling*.

[30] Batista C. R. A., Gomes G. F., Candelario-Jalil E., Fiebich B. L., de Oliveira A. C. P. (2019). Lipopolysaccharide-induced neuroinflammation as a bridge to understand neurodegeneration. *International journal of molecular sciences*.

[31] Eun B. L., Abraham J., Mlsna L., Kim M. J., Koh S. (2015). Lipopolysaccharide potentiates hyperthermia-induced seizures. *Brain and Behavior: A Cognitive Neuroscience Perspective*.

[32] Shin E. J., Nah S. Y., Kim W. K. (2005). The dextromethorphan analog dimemorfan attenuates kainate-induced seizures *via* sigma 1 receptor activation: comparison with the effects of dextromethorphan. *British Journal of Pharmacology*.

[33] Yang L. X., Chen F. Y., Yu H. L. (2020). Poncirin suppresses lipopolysaccharide (LPS)-induced microglial inflammation and ameliorates brain ischemic injury in experimental stroke in mice. *Annals of Translational Medicine*.

[34] Stahl S. M. (2019). Dextromethorphan/bupropion: a novel oral NMDA (N-methyl-d-aspartate) receptor antagonist with multimodal activity. *CNS Spectrums*.

[35] Keller M., Griesmaier E., Auer M. (2008). Dextromethorphan is protective against sensitized N-methyl-D-aspartate receptor-mediated excitotoxic brain damage in the developing mouse brain. *The European Journal of Neuroscience*.

[36] Yang P. P., Huang E. Y. K., Yeh G. C., Tao P. L. (2006). Co-administration of dextromethorphan with methamphetamine attenuates methamphetamine-induced rewarding and behavioral sensitization. *Journal of Biomedical Science*.

[37] Hung C. C., Lee Y. H., Kuo Y. M. (2019). Soluble epoxide hydrolase modulates immune responses in activated astrocytes involving regulation of STAT3 activity. *Journal of Neuroinflammation*.

[38] Li D., Liu X., Liu T. (2020). Neurochemical regulation of the expression and function of glial fibrillary acidic protein in astrocytes. *Glia*.

[39] Brenner M. (2014). Role of GFAP in CNS injuries. *Neuroscience Letters*.

[40] Sardinha V. M., Guerra-Gomes S., Caetano I. (2017). Astrocytic signaling supports hippocampal-prefrontal theta synchronization and cognitive function. *Glia*.

[41] Colle D., Santos D. B., Naime A. A. (2020). Early postnatal exposure to paraquat and maneb in mice increases nigrostriatal dopaminergic susceptibility to a re-challenge with the same pesticides at adulthood: implications for Parkinson's disease. *Neurotoxicity Research*.

[42] Gogal R. M., Holladay S. D. (2008). Perinatal TCDD exposure and the adult onset of autoimmune disease. *Journal of Immunotoxicology*.

[43] Takano T., Tian G. F., Peng W. (2006). Astrocyte-mediated control of cerebral blood flow. *Nature Neuroscience*.

[44] Straub S. V., Nelson M. T. (2007). Astrocytic calcium signaling: the information currency coupling neuronal activity to the cerebral microcirculation. *Trends in Cardiovascular Medicine*.

[45] Chechneva O. V., Mayrhofer F., Daugherty D. J., Pleasure D. E., Hong J. S., Deng W. (2011). Low dose dextromethorphan attenuates moderate experimental autoimmune encephalomyelitis by inhibiting NOX2 and reducing peripheral immune cells infiltration in the spinal cord. *Neurobiology of Disease*.

[46] Zhang W., Wang T., Qin L. (2004). Neuroprotective effect of dextromethorphan in the MPTP Parkinson's disease model: role of NADPH oxidase. *The FASEB Journal*.

[47] Church J., Sawyer D., McLarnon J. G. (1994). Interactions of dextromethorphan with the N-methyl-d-aspartate receptor-channel complex: single channel recordings. *Brain Research*.

[48] Shioda N., Ishikawa K., Tagashira H., Ishizuka T., Yawo H., Fukunaga K. (2012). Expression of a truncated form of the endoplasmic reticulum chaperone protein, *σ*1 receptor, promotes mitochondrial energy depletion and apoptosis. *The Journal of Biological Chemistry*.

[49] Lockhart B. P., Soulard P., Benicourt C., Privat A., Junien J. L. (1995). Distinct neuroprotective profiles for *σ* ligands against N-methyl-d-aspartate (NMDA), and hypoxia-mediated neurotoxicity in neuronal culture toxicity studies. *Brain Research*.

[50] Vagnerova K., Hurn P. D., Bhardwaj A., Kirsch J. R. (2006). Sigma 1 receptor agonists act as neuroprotective drugs through inhibition of inducible nitric oxide synthase. *Anesthesia & Analgesia*.

